# Concerned Family Members’ Help-Seeking in Elder Family Financial Exploitation

**DOI:** 10.1093/geroni/igaa057.2147

**Published:** 2020-12-16

**Authors:** Tina Kilaberia, Marlene Stum

**Affiliations:** 1 UC Davis Health, Sacramento, California, United States; 2 University of Minnesota, Twin Cities, Saint Paul, Minnesota, United States

## Abstract

This paper examines non-perpetrator family members’ experience of trying to help when faced with elder family financial exploitation. Utilizing data from a qualitative study of 28 Concerned Family Members (CFMs) who were primarily adult children of older victims, findings provide evidence of the critical role CFMs play in helping the victims. Six help-seeking tasks are identified, including gathering evidence, learning new systems, and taking on money management roles. CFMs often put the victim’s health and well-being before their own, becoming secondary victims in the process. CFMs experienced a wide range of costs to their individual health and well-being, including physical, emotional, psychological, social and financial dimensions (e.g. stress, depression, inability to sleep, isolation, harassment, threats to personal safety, physical abuse, attorney and court costs, time off work). Findings have implications for supporting CFMs and addressing practical and health-related needs as secondary victims.

